# Greenland-wide inventory of ice marginal lakes using a multi-method approach

**DOI:** 10.1038/s41598-021-83509-1

**Published:** 2021-02-24

**Authors:** Penelope How, Alexandra Messerli, Eva Mätzler, Maurizio Santoro, Andreas Wiesmann, Rafael Caduff, Kirsty Langley, Mikkel Høegh Bojesen, Frank Paul, Andreas Kääb, Jonathan L. Carrivick

**Affiliations:** 1 Asiaq Greenland Survey, Nuuk, Greenland; 2 Gamma Remote Sensing, Gümligen, Switzerland; 3 Department of Geography, University of Zurich, Zurich, Switzerland; 4 Department of Geosciences, University of Oslo, Oslo, Norway; 5 School of Geography and Water@Leeds, University of Leeds, Leeds, UK; 6 DHI GRAS, Hørsholm, Denmark

**Keywords:** Cryospheric science, Hydrology

## Abstract

Ice marginal lakes are a dynamic component of terrestrial meltwater storage at the margin of the Greenland Ice Sheet. Despite their significance to the sea level budget, local flood hazards and bigeochemical fluxes, there is a lack of Greenland-wide research into ice marginal lakes. Here, a detailed multi-sensor inventory of Greenland’s ice marginal lakes is presented based on three well-established detection methods to form a unified remote sensing approach. The inventory consists of 3347 ($\pm 8$%) ice marginal lakes ($>0.05\,{{\text{ km }}^{2}}$) detected for the year 2017. The greatest proportion of lakes lie around Greenland’s ice caps and mountain glaciers, and the southwest margin of the ice sheet. Through comparison to previous studies, a $\sim 75$% increase in lake frequency is evident over the west margin of the ice sheet since 1985. This suggests it is becoming increasingly important to include ice marginal lakes in future sea level projections, where these lakes will form a dynamic storage of meltwater that can influence outlet glacier dynamics. Comparison to existing global glacial lake inventories demonstrate that up to 56% of ice marginal lakes could be unaccounted for in global estimates of ice marginal lake change, likely due to the reliance on a single lake detection method.

## Introduction

The Greenland Ice Sheet (GrIS) contains a considerable amount of the world’s fresh water resources, with its mass loss raising sea levels by 13.7 mm since 1979 and a possible contribution of $\sim 70$–126 mm by 2100^1–4^. A large amount of the GrIS drains to a terrestrial margin, where meltwater can form large reservoirs that delay the outflow of meltwater to the ocean and alter its biogeochemistry. This is also understood to buffer melt contribution to the sea level budget, with meltwater partially being stored on land in endorheic reservoirs^5–8^.

Ice marginal lakes form a dynamic component of terrestrial meltwater storage^9^. Proglacial lakes (including ice marginal lakes) currently hold up to 0.43 mm of sea level equivalent globally, which remains unaccounted for in present sea level change estimates^10^. Ice marginal lakes form at the fringes of glaciers and ice sheets where the outflow is dammed or restricted; for instance, by the ice itself or a moraine. Ice marginal lakes can burst and cause catastrophic flooding when the water level in these lakes reaches a critical level or the lake dam fails^11–14^. Such events are known as jökulhlaups (the Icelandic term) or Glacial Lake Outburst Floods (GLOFs). Beside this natural hazard potential for local residents and infrastructure, GLOFs can drastically affect the downstream landscape and ecosystems^15^ through abrupt influxes of suspended sediment^16^, water salinity changes^17^, and enhanced erosion and deposition^18–20^. For example, the large flux of sediment and freshwater from GLOF events at Russell Glacier, SW Greenland, have been known to disrupt fisheries downstream near the settlement of Kangerlussuaq^21^.

Recent studies have indicated that the number of ice marginal lakes in Greenland has increased over the past three decades, inundating larger areas of the terrestrial landscape^10,22^. In turn, the dynamics of GLOF events have also changed, for example GLOF frequency and GLOF water routing^19,23,24^. Changes in Greenland’s ice marginal lakes will undoubtedly have repercussions for future sea level, with future GrIS melt predicted to cause GLOFs that have the potential for mega-flood type impacts^14^. It is therefore of paramount importance to monitor ice marginal lakes to better understand the future impacts on Greenland’s terrestrial and marine landscapes, ecosystems, and human activities (e.g. hydropower and tourism). In order to adequately monitor ice marginal lake change, a Greenland-wide inventory is needed to provide a baseline for a related change assessment.

In spite of focused research on individual ice marginal lakes and regional studies, there is currently a lack of Greenland-wide research into ice marginal lakes. Lake changes have previously been monitored in detail over small areas using in situ measurements^11^ and remote sensing^25,26^, along with forecast modelling to predict future dynamics^13^. Remote sensing approaches have also proved advantageous for monitoring water bodies over large regions of Greenland, such as spectral indices generation from optical and infrared imagery^27^, classification from radar imagery^28^, and sink detection from Digital Elevation Models (DEMs)^29^. However, each of these remote sensing approaches has known limitations. For example, ice cover on lakes is understood to limit classification from optical and SAR imagery^28^. Therefore, reliance on a single approach can introduce uncertainty through mis-classification, or underestimation^30^. An ensemble approach that combines these methods is essential to successful classification of ice marginal lakes over whole regions with a high degree of certainty^31^.

This study presents a comprehensive, Greenland-wide inventory of ice marginal lakes for the year 2017 using a multi-sensor and multi-method approach. Three well-established approaches were used to classify water bodies: (1) multi-temporal backscatter classification using Sentinel-1 synthetic aperture radar (SAR) imagery (hereafter referred to as S1); (2) multi-spectral indices classification using Sentinel-2 optical imagery (S2); and (3) sink detection using the ArcticDEM (ADEM). The results from these approaches were subsequently compiled and quality-checked to produce the 2017 Inventory of Ice Marginal Lakes (IIML).

## Results

### Inventory overview

**Figure 1:**
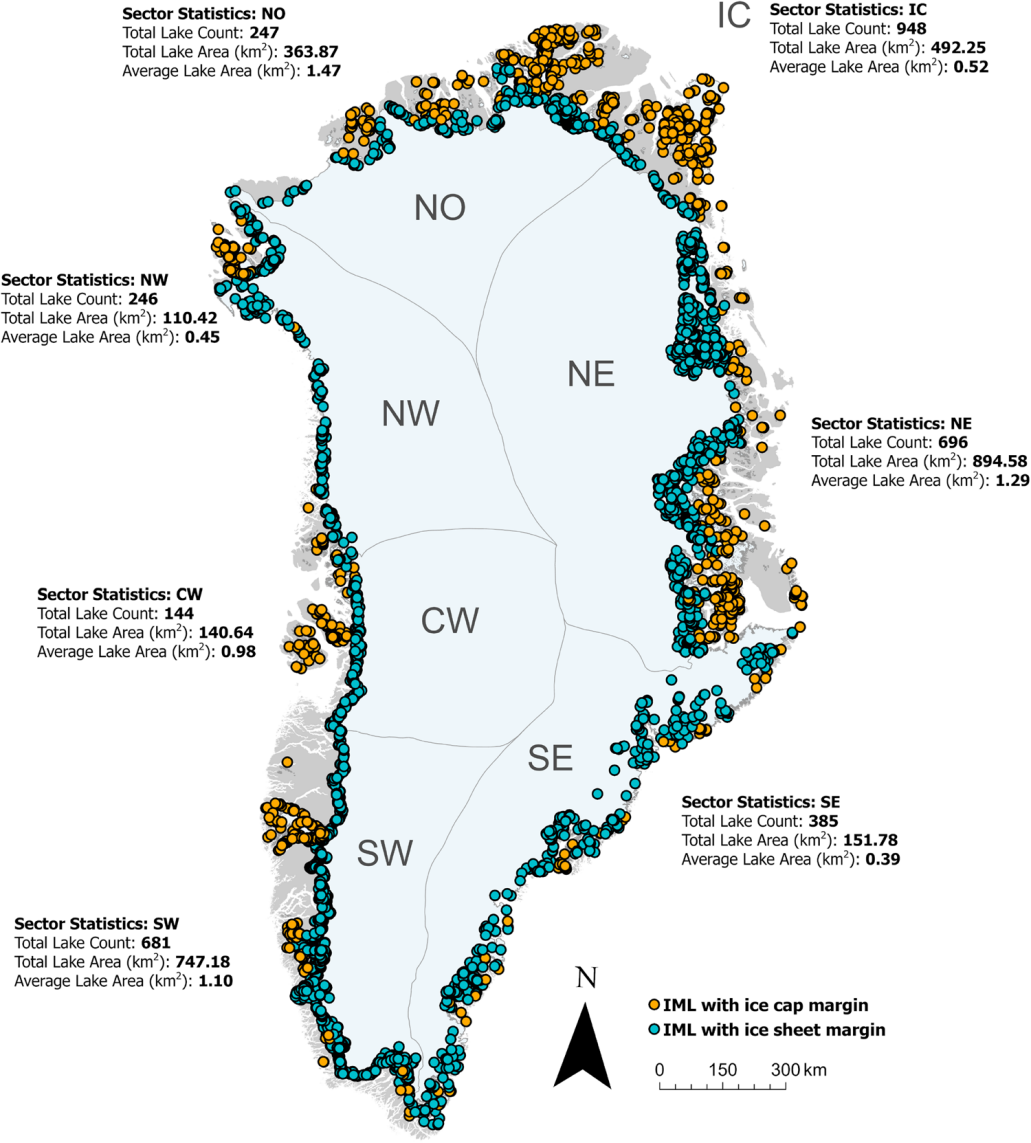
Overview of the 2017 ice marginal lake inventory of Greenland, where each defined point represents one unique ice marginal lake. Ice sheet basins are based on those classified as ice catchments by Mouginot and Rignot^32^, with blue points denoting lakes sharing a margin with the ice sheet. Ice marginal lakes adjacent to Greenland’s ice caps and mountain glaciers are those points in orange, corresponding to the sector statistics (IC). Figure generated with ArcGIS Pro (v2.6.1, https://www.esri.com/en-us/arcgis/products/arcgis-pro/)^66^.

Overall, 4530 polygon features were detected with many overlapping and corresponding to the same ice marginal lake under the combination of the three independent detection methods. Disregarding multiple counting of overlapping polygons, the IIML indicates that there were 3347 ($\pm 8$%) unique ice marginal lakes above a minimum area of $0.05\,{{\text{ km }}^{2}}$ (derived as an average of overlapping polygons) in Greenland in 2017 (Fig. 1). The inventory consists of lakes formed at the ice sheet margin and the margin of Greenland’s peripheral ice caps and mountain glaciers. This also includes lakes formed around nunataks within 1 km of the ice sheet margin, based on a modified version of the MEaSUREs GIMP (Greenland Ice Mapping Project) 15 m ice mask (see “Methods” section for more details). A large majority of ice marginal lakes in the inventory are nameless, with 3194 (95%) unnamed lakes in the IIML based on the Language Secretariat of Greenland (Oqaasileriffik) placename database.

**Figure 2:**
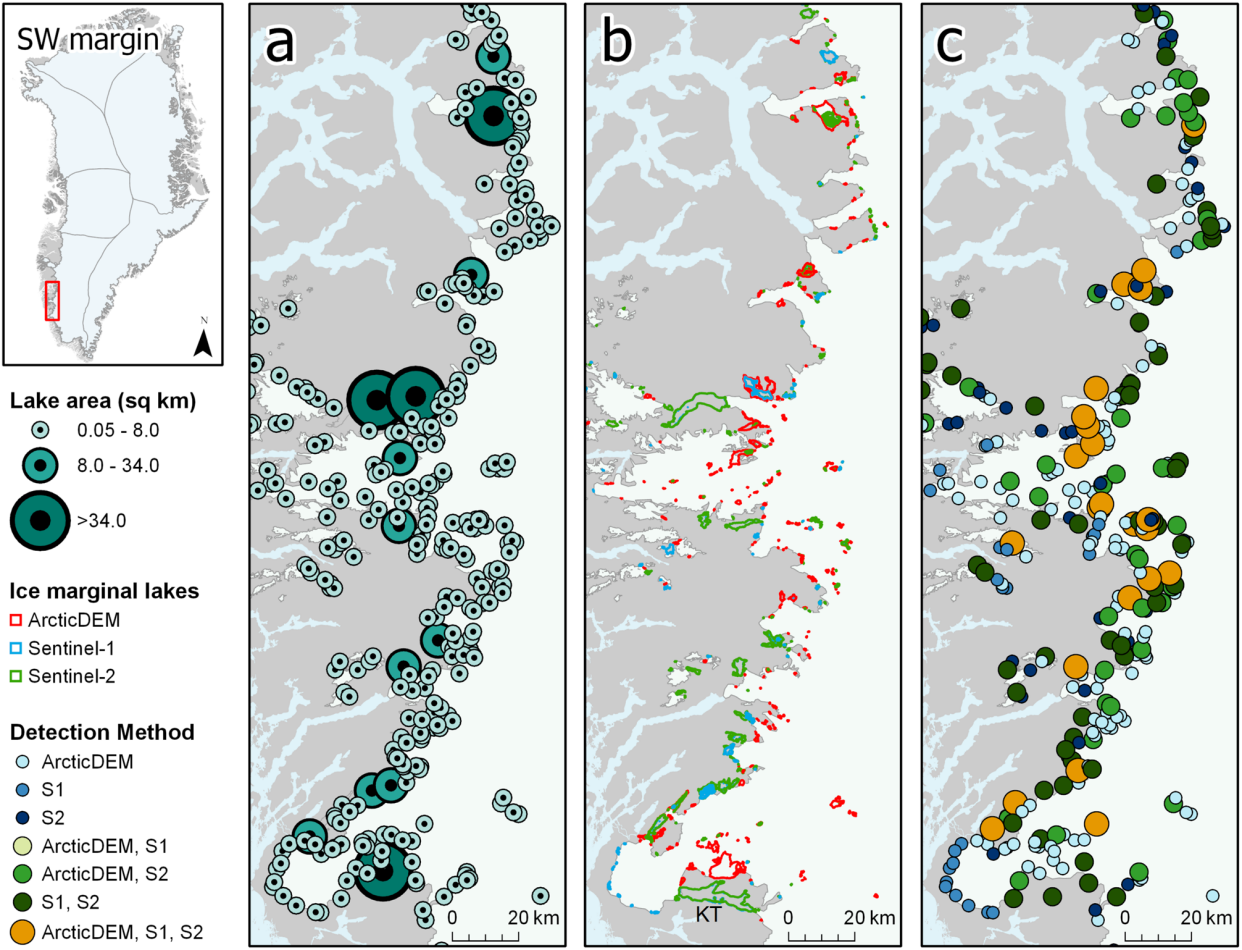
Ice marginal lakes over a selected section of the SW ice sheet margin, where (**a**) lake area, (**b**) lake shape determined by each method (as described in the “Methods” section), and (**c**) detection method are presented. Ice, land and ocean are displayed in white, grey and light blue, respectively. The ice margin shown is a modified version of the MEaSUREs GIMP ice mask^33^. The largest lake of this region (Kangaarssuup Tasersua) is labelled as KT in **b**. Figure generated with ArcGIS Pro (v2.6.1, https://www.esri.com/en-us/arcgis/products/arcgis-pro/)^66^.

The highest number of ice marginal lakes are generally present at the longest land-terminating sections of the GrIS, namely the southwest margin (SW, Fig. 1) and the northeast margin (NE), and the surrounding ice caps and mountain glaciers (IC). Ice marginal lakes are most abundant around the IC sector, accounting for 28% of the inventory (948 ice marginal lakes). The SW margin is the most densely populated section of the ice sheet margin for ice marginal lakes with an average spacing of 5.85 km between each lake (Fig. 2), and includes the fourth largest of the inventory, Kangaarsuup Tasersua (KT, Fig. 2b).

The least number of ice marginal lakes occur along the central west margin (CW), with only 144 lakes detected; typically forming in the proglacial area or at the lateral margins of ice sheet outlets such as Eqip Sermia, Store Glacier (also known as Sermeq Kujalleq) and Lille Glacier (also known as Sermeq Avannarleq) (Fig. S1). Despite the southeast (SE) being the longest margin at 14,911 km, it is one of the least lake-populated sections with only 385 ice marginal lakes at an average distancing of 39 km.

**Figure 3:**
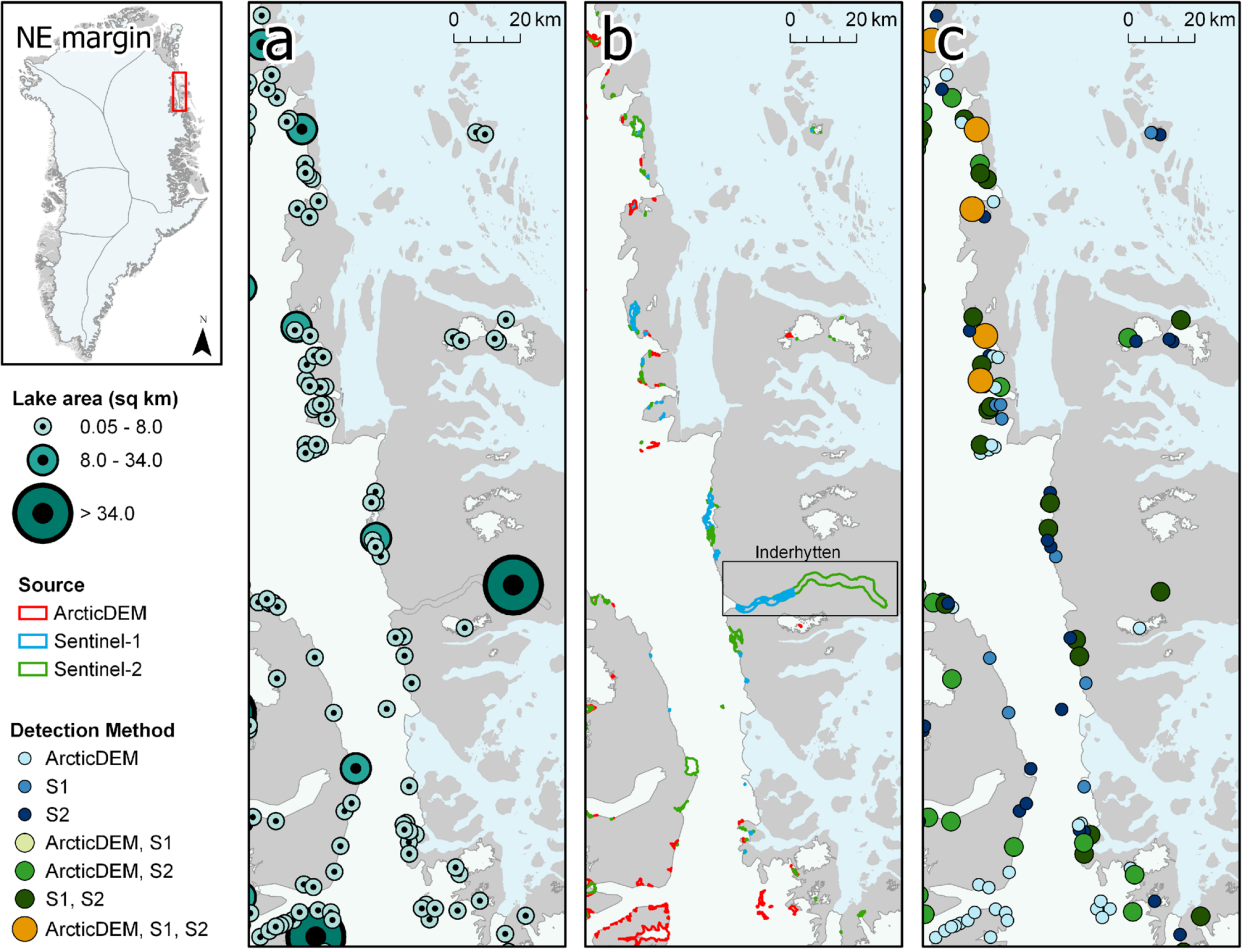
Ice marginal lakes over a selected section of the NE ice sheet margin, where (**a**) lake area, (**b**) lake shape determined by each method (as described in the “Methods” section), and (**c**) detection method are presented, including the second largest lake of the IIML, Inderhytten (black box). Ice, land and ocean are displayed in white, grey and light blue, respectively. The ice margin shown is a modified version of the MEaSUREs GIMP ice mask^33^. Figure generated with ArcGIS Pro (v2.6.1, https://www.esri.com/en-us/arcgis/products/arcgis-pro/)^66^.

Ice marginal lakes are typically smaller than $0.5\,{{\text{ km }}^{2}}$, with 2663 lakes (80%) falling within a range between 0.05 and $0.5\,{{\text{ km }}^{2}}$ and only 424 lakes larger than $1.00\,{{\text{ km }}^{2}}$ (Fig. S2). The largest named ice marginal lake of the 2017 inventory is Romer Sø ($130.87\,{{\text{ km }}^{2}}$), where the piedmont glacier Elephant Foot Glacier terminates. The second largest ice marginal lake is Inderhytten ($112.02\,{{\text{ km }}^{2}}$), a substantial lake at the terminus of Sælsøgletsjer at the NE margin (Fig. 3). The third largest is an unnamed lake ($91.58\,{{\text{ km }}^{2}}$) along the SW margin, approximately 100 km south of the settlement of Kangerlussuaq. The largest ice marginal lakes are generally found in the northern region of the ice sheet margin, with an average area of $1.47\,{{\text{ km }}^{2}}$ ($0.23\,{{\text{ km }}^{2}}$ median; $5.76\,{{\text{ km }}^{2}}$ standard deviation., Table S1) along the north margin (NO), and an average area of $1.29\,{{\text{ km }}^{2}}$ ($0.22\,{{\text{ km }}^{2}}$ median; $4.00\,{{\text{ km }}^{2}}$ standard deviation, Table S1) along the NE margin.

### Performance of the methodologies

**Figure 4:**
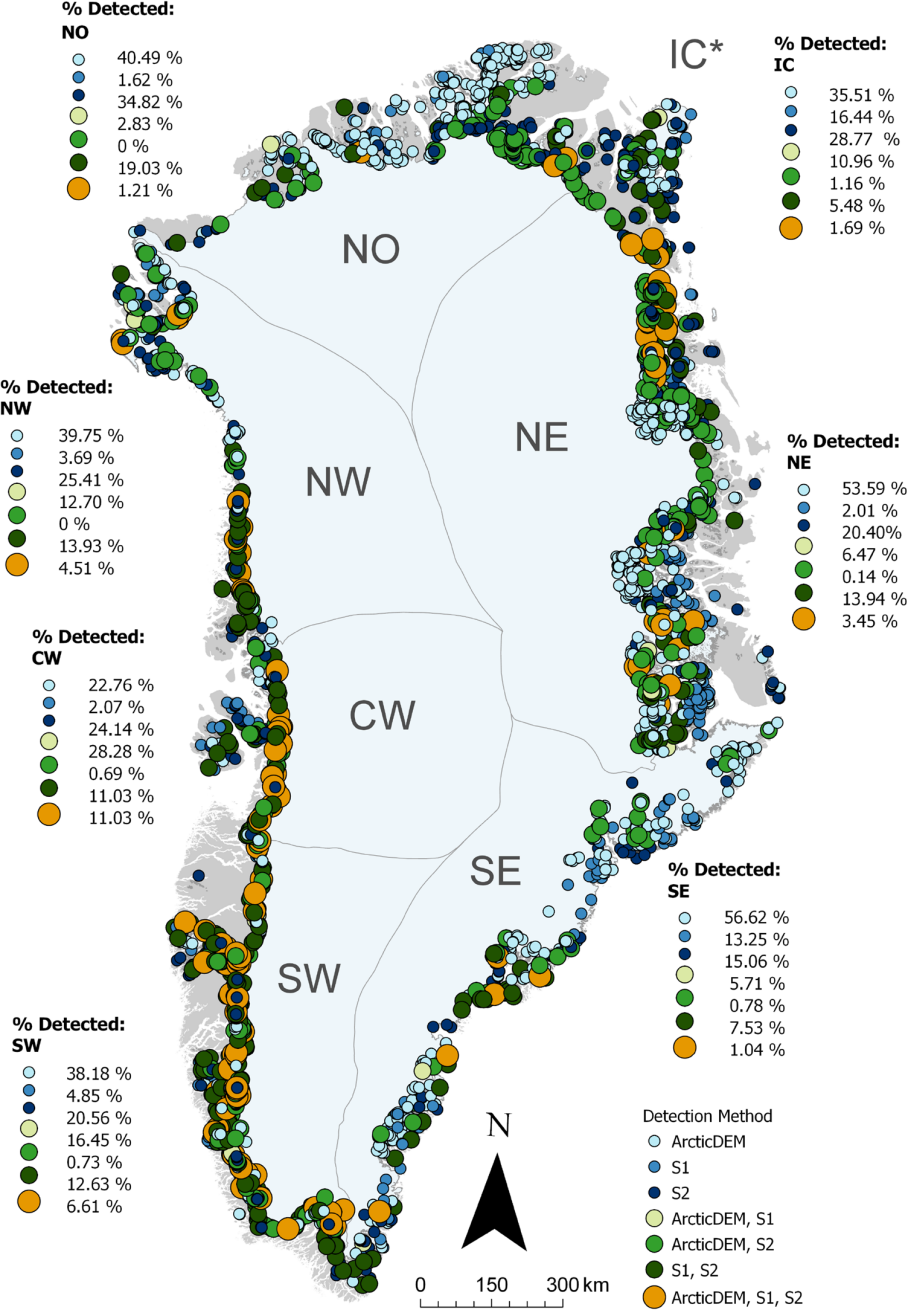
The number of detection methods that successfully classified each ice marginal lake, split by region with percentage breakdowns of each combination of detection methods (see Table S1 for further details). Ice sheet basins are based on those classified as ice catchments by Mouginot and Rignot^32^. Figure generated with ArcGIS Pro (v2.6.1, https://www.esri.com/en-us/arcgis/products/arcgis-pro/)^66^. *IC denotes ice marginal lakes found at the margins of Greenland’s ice caps and mountain glaciers.

The majority (74%) of the polygons in the IIML were identified using only one method, with over half of these instances being ADEM-only (Table S2). Where an ice marginal lake was detected using two methods (22%), S2 polygons were generally detected along with another method (making up 723 of 744 of these instances). There are few polygons detected from both ADEM and S1 (21 lakes), which is likely to reflect the larger number of S2- and ADEM-derived ice marginal lakes in the IIML. Only 199 ice marginal lakes (4%) were detected using all three methods. Successful identification with all three methods appears to have no visible correlation with lake form or size, but varies according to each section of the GrIS margin, with the most effective detection occurring along the SW and CW margins (Fig. 4).

From examining the outlines where ice marginal lakes were detected successfully with all three methods, S1- and S2-derived lakes unsurprisingly had the smallest difference in extent given that these methods detect water presence directly, with an average areal difference of $0.14\,{{\text{ km }}^{2}}$ (Table S3). Larger areal differences are evident when comparing the ADEM-derived lakes to the S1- and S2-derived lakes, with an average difference of $0.36\,{{\text{ km }}^{2}}$ (64%) and $0.25\,{{\text{ km }}^{2}}$ (49%), respectively. Taking the maximum and minimum detected extents for each lake, this produces an average area difference of $0.37\,{{\text{ km }}^{2}}$, equating to an areal range of 70% for each lake. This suggests that the number of methods that successfully identified an ice marginal lake can be used as a measure of certainty, where ice marginal lakes detected from all three methods (ADEM, S1, S2) denote the highest level of certainty in lake presence. However, this does not necessarily reflect the lake outline accuracy, given that the outlines are derived from different time steps.

## Discussion

### Multi-sensor detection methods are needed to encompass latitudinal range of Greenland

Successful identification of ice marginal lakes using all three methods used in this study varies according to region, with most effective detection occurring at the SW and CW margins (Fig. 4). Of the 681 ice marginal lakes identified at the SW margin, 248 were detected through two or more sources (36%); as were 73 of the 144 lakes at the CW margin (i.e. 51%) (Table S1). This could be indicative of optimum conditions for detecting ice marginal lakes (e.g. lack of ice cover) in these regions compared to others.

Other regions are largely composed of lakes detected with just a single source, such as the high abundance of ADEM-only detected lakes at the NO margin (100 lakes, 40%) and the NE margin (373 lakes, 54%) (Fig. 4, Table S1). A strength of the ADEM method compared to the S1 and S2 methods is detecting lake basins where the lake is either partially or completely covered with ice^29^. This is particularly important in the high-latitude regions or lakes within a topography with a northern aspect, where ice cover is known to persist throughout the melt season. Such areas are understood to be notoriously challenging for lake classification from optical and SAR imagery due to persistent ice cover, even during the summer season^10,34^. The IIML results reflect this, with the difference in successful lake classification across each region indicative of the large latitudinal range of climatic conditions along Greenland. The inclusion of the ADEM method in this study is therefore crucially important, with ADEM-derived lakes making up nearly half of all the lakes detected across the northern region. These high latitude lakes would otherwise be unaccounted for in optical and/or SAR-derived lake classifications, and would result in a marked under-representation of ice marginal lakes in Greenland.

### Increasing ice marginal lake abundance in West Greenland

**Figure 5:**
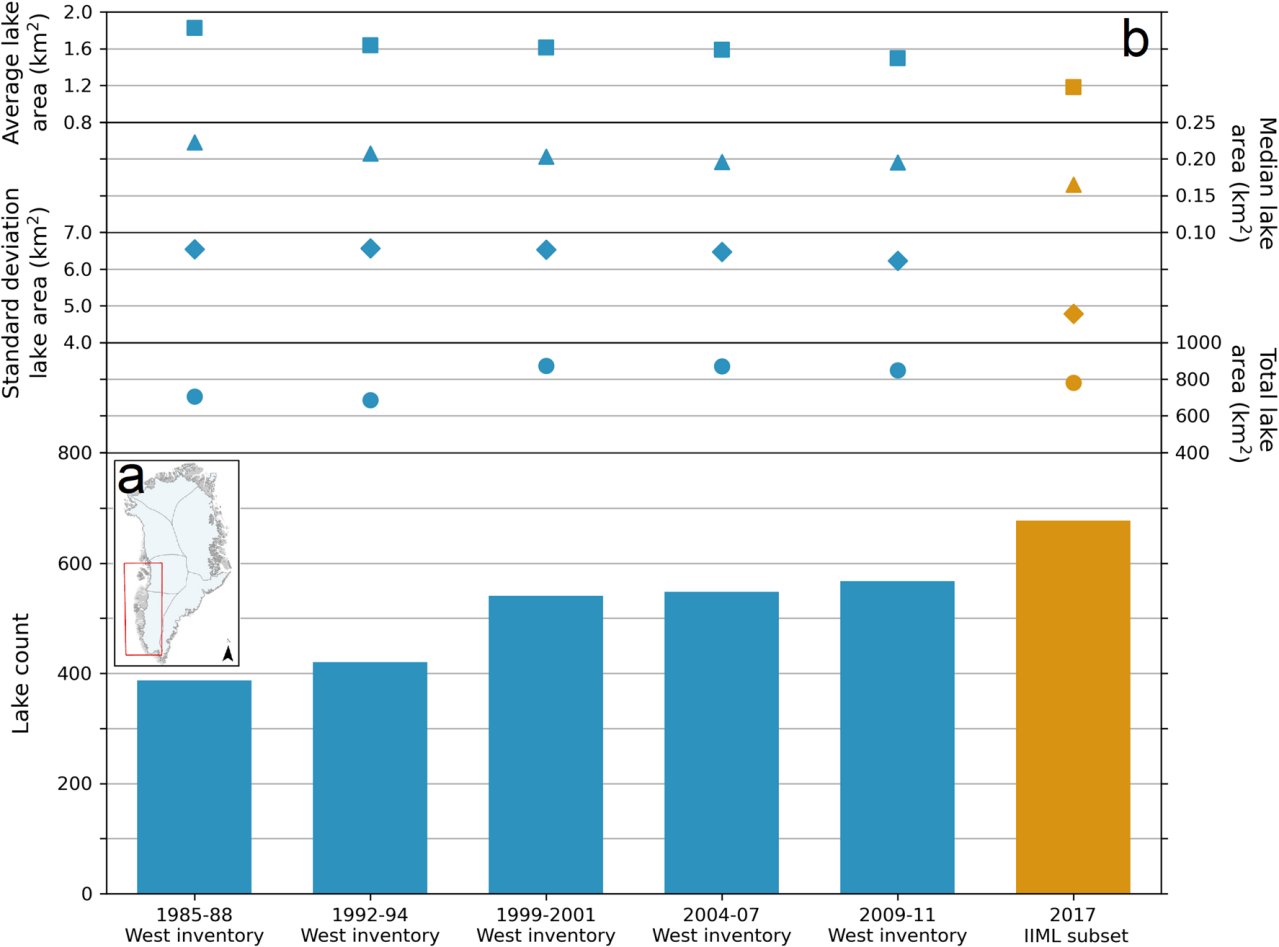
Change in ice marginal lakes along the west margin of the GrIS, where (**a**) shows the west margin sector and (**b**) the ice marginal lake time-series from 1985 to 2017. The square point plot (top) denotes average lake area, the circle point plot (middle) shows total lake area, and the bar plot (bottom) depicts lake count. All inventories between 1985–1988 and 2009–2011 (blue) are as presented by Carrivick and Quincey^22^. Data points from 2017 are a subset taken from the IIML presented in this study (orange). The subset covers the same sector of the west margin, where the S1- and S2-derived lakes are preferenced as alike methods to the Landsat-derived lakes, based on the principle of lake inference from water presence. The 2017 values presented are an average of the subset with and without ADEM-only lakes, thereby preferencing S1- and S2-derived lakes, but still including lakes identified using the ADEM method.

The 2017 IIML represents ice marginal lakes for a discrete time period. The dynamical change of these ice marginal lakes can be better explored when compared to pre-existing datasets, such as those derived from optical Landsat 4–8 imagery by Carrivick and Quincey^22^ for selected years between 1985 and 2011 (1985–1987, 1992–1994, 1999–2001, 2004–2007, and 2009–2011) over the SW and CW margin of the GrIS (excluding IC) (Fig. 5a). A subset of the IIML covering the same study region is presented for the purpose of this comparison, weighted to the S1- and S2-derived lakes (as they are detection methods based on the principle of lake inference from water presence, as adopted by Carrivick and Quincey^22^). This subset generally reflects the method breakdown of the entire IIML, with 60% of lakes classified using one method and 40% derived from two or three methods.

A total of 387 ($\pm 6.5$%) ice marginal lakes were identified along the west margin in the 1985–1988 inventory, whilst 678 ($\pm 8$%) lakes have been identified in the 2017 IIML; suggesting a $\sim 75$% increase in the number of lakes along the west margin over the past three decades (Fig. 5b). Minimal changes in total lake area are evident along the west margin. However, there is a decreasing trend in individual lake area, with average area decreasing from 1.82$\,{{\text{ km }}^{2}}$ (1985–1988) to 0.95$\,{{\text{ km }}^{2}}$ (2017) and median area decreasing from 0.22$\,{{\text{ km }}^{2}}$ (1985–1988) to 0.16$\,{{\text{ km }}^{2}}$ (2017). This trend reflects marked variations in the abundance of small lakes (i.e. 0.05–0.15$\,{{\text{ km }}^{2}}$), and is suggested to be a possible explanation for the trend in total lake area (Fig. S3). The spatial resolution of these datasets differ—Carrivick and Quincey^22^ primarily used 30 m Landsat imagery whereas the IIML uses data with a spatial resolution ranging from 5 to 10 m. However, the trend in lake abundance evident in this comparison is unlikely to be directly attributed to a difference in spatial resolution, given that the minimum lake area is 0.05$\,{{\text{ km }}^{2}}$ in both these records. This comparison therefore suggests an increase in lake abundance across the west margin of the GrIS.

Carrivick and Quincey^22^ proposed that the changes between 1987 and 2010 were inherently linked to the 0.8% year^-1^ mean percentage change in ice sheet surface melt^35^. The results from the 2017 IIML suggest that increasing lake abundance likely reflects the enhanced retreat of the ice margin, with formation occurring in front of retreating outlets. Future lake formation is likely to be concentrated in regions where marine-terminating outlets retreat on to land, as the terrestrial margin length will increase and hold a sinuous form^36^. Ice marginal lakes will therefore form a crucial component in the dynamics of these outlets during this transitional phase^37,38^. Additionally, the dynamics of Greenland’s terrestrial store of freshwater will alter if this trend of increasing lake abundance with decreasing size continues into the future. This could possibly influence the transfer of freshwater from the GrIS to the ocean, not only affecting melt contribution to the sea level budget, but also with likely effects on Greenland’s freshwater resources, ecosystems and biogeochemical fluxes^39,40^.

### High sensitivity in records of remotely-sensed ice marginal lake change

The multi-method approach to this study has shown how the choice of method strongly impacts the number of lakes detected; for instance, 49% of the IIML is derived using the S2 optical method alone. Recent studies into glacial lake change have relied solely on optical classification approaches, such as the global inventory of glacial lakes presented by Shugar et al.^10^ The glacial lake inventory presented by Shugar et al.^10^ (including ice caps and ice sheets, and consisting of ice marginal, recently detached proglacial, and near-terminus supraglacial lakes) implemented a NDWI and NDSI (Normalised Difference Snow Index) approach to classify lakes within a 1 km buffer of the ice margin. The glacial lake inventory suggested an overall increase of 53% in the number of lakes between 1990–1999 (9414 lakes) and 2015–2018 (14,393 lakes), with a total area growth of 51%. Of those glacial lakes detected in Greenland in 2015–2018, only 44% corresponded with ice marginal lakes from the current study (the IIML), suggesting that up to 56% of ice marginal lakes in Greenland remain unaccounted for in current global estimates. Whilst this could be a result of the differing resolutions of the IIML (10 m spatial resolution for 2017) and the glacial lake inventory from Shugar et al.^10^ (30 m spatial resolution for 2015–2018), it is also likely that the wider range of classification approaches used in the IIML results in better lake identification under varying environmental and imaging conditions, such as the challenges with cloud cover in optical satellite scenes, ice-covered lakes, and lake water turbidity. This highlights a trade-off between classification accuracy and study feasibility, where multi-method implementations for producing global datasets demand more time and processing power at the expense of reduced accuracy.

Greenland-wide estimates of ice marginal lake extent are highly sensitive to mis-classifications. For example, Inderhytten is the second largest ice marginal lake in Greenland (as shown in Fig. 3) at 112.02$\,{{\text{ km }}^{2}}$ according to the IIML. However, Inderhytten is not included in the glacial lake inventory presented by Shugar et al.^10^ because it is ice covered for the majority of the year and lies at an elevation below the threshold of their classification ($<5$ m a.s.l.). The absence of Inderhytten would modify the dataset substantially if left out of the IIML, skewing the average lake size and total area of the dataset by 4%. Such a substantial impact on Greenland-wide estimates not only draws attention to the problem of mis-classifications, but also demonstrates the potential of implementing multi-sensor and multi-method approaches in lake detection to reliably and accurately define ice marginal lake change.

## Conclusions

The Greenland-wide inventory of ice marginal lakes uncovers 3347 ($\pm 8$%) unique lakes (above 0.05$\,{{\text{ km }}^{2}}$) in 2017, using a multi-method approach incorporating backscatter classification from Sentinel-1 satellite imagery, multi-spectral indices classification from Sentinel-2 satellite imagery, and sink detection from the high-resolution ArcticDEM. The average lake size of the entire inventory is 0.88$\,{{\text{ km }}^{2}}$ with the largest being Romer Sø (130.87$\,{{\text{ km }}^{2}}$), situated on the NE margin of the GrIS. A high number of lakes are around Greenland’s ice caps and mountain glaciers, and along the SW margin of the ice sheet, collectively accounting for nearly half (49%) of the inventory.

The multi-method approach provides an effective means of evaluating the certainty of each detected ice marginal lake. Overall, 26% of the inventory was identified with two or more methods, with a high majority of those identified using the sink detection method. Successful identification with all three methods has no correlation with lake form or size, but does appear to be region-dependent with the most effective detection occurring along the SW and CW margins. This likely reflects the optimum climatic conditions in these regions, such as the lack of summer ice cover.

Greenland-wide estimates of ice marginal lake change are impeded by method limitations in remote sensing, which can lead to mis-classifications and under-representation of lakes. Comparison to a recent global glacial lakes inventory suggests that over half of ice marginal lake changes could be unaccounted for in current global estimates, with only 44% of lakes from the IIML (presented in this study) accounted for in a global glacial lake inventory^10^. The lake detection and filtering methods have to be carefully selected and might not be applicable to all regions in the same combination. For Greenland, only the multi-sensor and multi-method approaches applied here provided satisfactory results and a solid baseline dataset for change assessment. This highlights the power and potential that the increasing availability of high-resolution global satellite remote sensing datasets from different sensors (optical, radar, stereo etc.) and the improving computational possibilities to process and analyse such big amounts of data offers for environmental mapping and monitoring in general.

Lake change analysis along the west margin of the GrIS suggests a $\sim 75$% increase in the number of ice marginal lakes over the past three decades since 1985^22^. This trend likely follows increases in melt runoff and the retreating ice margin, with new lake formation occurring in front of retreating outlets. This suggests that ice marginal lakes will be of growing importance to the terrestrial store of water in Greenland. Glacier dynamics and mass balance are also influenced by lake termination, hence lake evolution can have significant effects on ice flow and melt and is not just a passive result of changes in ice margin position. Not only will ice marginal lakes likely be a significant dynamic component in future sea level contribution from the GrIS, but they will also have implications for freshwater resource management and ecosystems in Greenland which need to be examined in long-term monitoring strategies. Overall, this inventory provides a benchmark to conduct further analysis from, and develop our understanding of ice marginal lake dynamics on a Greenland-wide scale.

## Methods

### Sentinel-1 multi-temporal backscatter classification

Open permanent water bodies were identified from Sentinel-1 SAR images acquired during 2017. Over Greenland, Sentinel-1 was operated in the Interferometric Wide Swath (IWS) mode at Horizontal–Horizontal (HH) and Horizontal–Vertical (HV) polarisation with a repeat-pass of 12 days. The SAR images were provided by the European Space Agency (ESA) in Ground Range Detected (GRD) format with a pixel size of 10 m. Each image was averaged to the original spatial resolution of the data (20 m) and calibrated to gamma0 using an ArcticDEM mosaic^41^. Each image was then transformed from the radar acquisition geometry to the map geometry using a geocoding look-up table, created using the orbital information, SAR image processing parameters, and the ArcticDEM mosaic^42,43^. The geocoded images were finally tiled to a predefined grid of $100 \times 100$ km large blocks for easier data handling. From the individual images of the SAR backscattered intensity, monthly averages per polarisation (*AVE*) were derived to overcome the issue of speckle noise in a single image:

1 $$\begin{aligned} AVE = 10 *{\text{ log }}\left( \frac{1}{N} \sum \limits _{i=1}^{N} I_i \right) \end{aligned}$$

where $I_i$ is the SAR backscatter, and *i* is between 1 and *N* (i.e. the number of SAR observations at a given pixel in a given month, for a given polarisation).

Water bodies were detected using an ensemble-bagged tree classifier applied to the set of 24 predictors consisting of the 12 monthly SAR backscatter average values for each polarization. Including the entire time series of observations in the classifier served to reduce water commission errors introduced by wet snow and ice conditions, in which case the SAR backscatter is similar to the level observed over open water^44^. The classifier was trained with samples extracted from a water classification of SPOT images covering the Disko Bay. Lacking a similar dataset for the rest of Greenland, it was assumed that the classification rules based on the Disko Bay area would be equally applicable throughout the country.

Classification errors were limited through a set of post-processing steps which included the removal of isolated pixels and polygons smaller than 15 pixels, and removal of water bodies located on slopes steeper than $10^{\circ }$ according to the ArcticDEM mosaic^44,45^. This 15-pixel threshold was determined from testing with increasing thresholds, where 15 pixels turned out to be the optimum value for adequate removal of false detection whilst preserving small positive classifications.

### Sentinel-2 multi-spectral indices classification

Standard TOA (Top-Of-Atmosphere) Sentinel-2 L1C scenes were used in this study because standard surface reflectance products were not available at the time of processing. The standard TOA scenes were used as atmospheric corrections over Greenland are complex and have a risk of introducing sources of error^46^. Scenes were automatically selected based on cloud cover ($< 50$%) over July and August 2017 in order to limit lake ice and snow cover and maximise successful water classification. These Sentinel-2 scenes were processed in the local UTM projection (UTM Zone 24N), and bands 11 and 12 were re-sampled from 20 to 10 m using a nearest neighbour approach.

**Figure 6:**
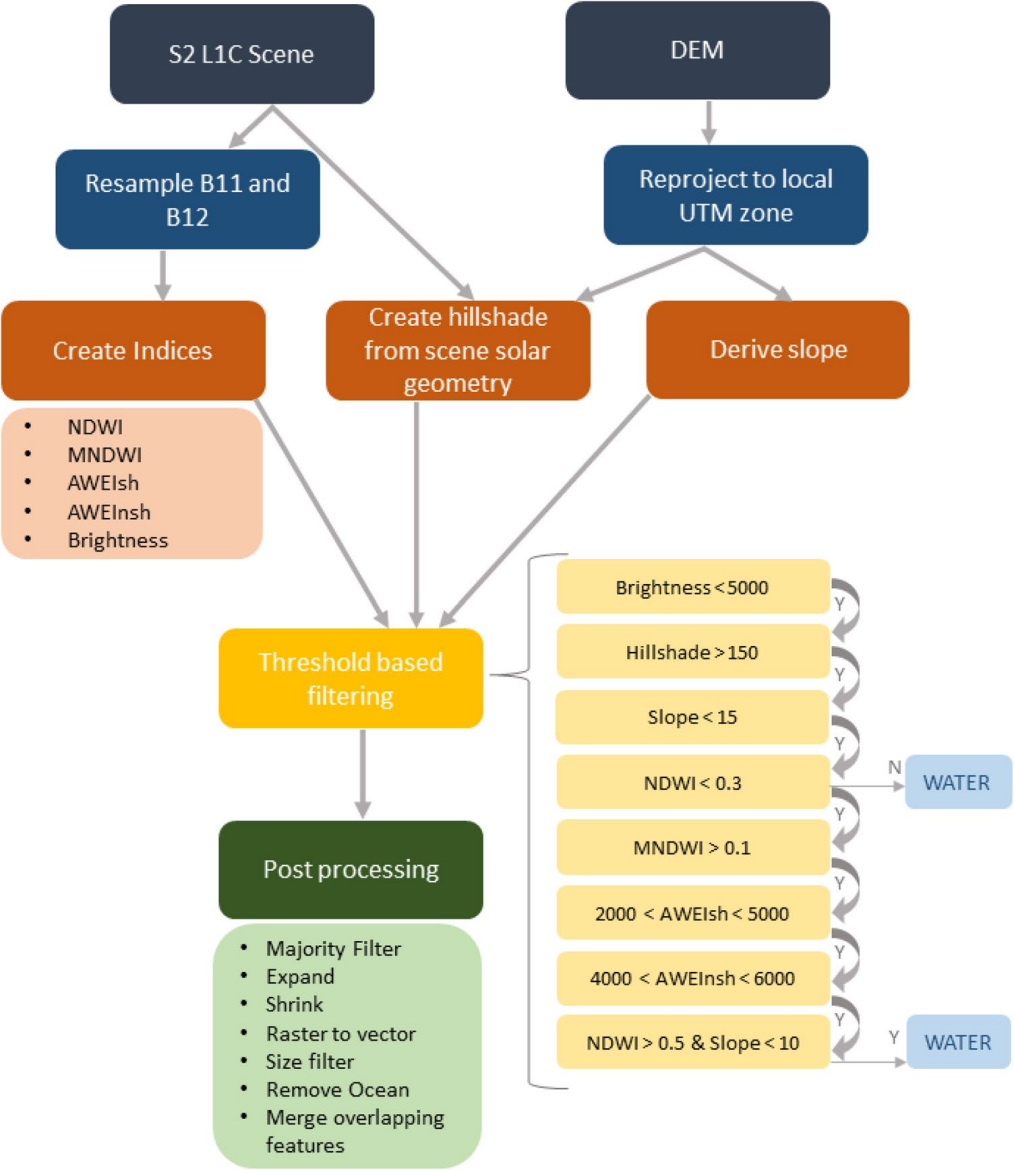
Flow diagram presenting the processing chain for classifying ice marginal lakes from Sentinel-2 imagery, including terrain indices generation using a DEM and multi-spectral indices thresholding, which is compiled through a decision tree classifier to extract water bodies.

Water bodies were classified using a multi-terrain and multi-spectral indices approach through the processing chain as demonstrated in Fig. 6^31,47^. The multi-terrain indices consists of a slope and aspect index calculated from sun geometry (available in the scene metadata) in relation to topography slope and aspect^48^. The multi-terrain indices distinguishes regions at risk to false positive detection caused by topographic shadowing and sun glint, and are subsequently masked out^31,49^.

The multi-spectral indices are a collection of well-established spectral indices—NDWI, MNDWI, AWEIsh, AWEInsh and brightness (Table 1)—which are passed through a multi-layer decision tree classification to classify water bodies (Fig. 6). A coarse brightness threshold (more typically referred to as grayscale transformation) is applied, which acts as a simple initial filter for removing regions where highly reflective snow and ice are present. The NDWI and MNDWI are highly effective at detecting optically clear water bodies not affected by shadowing^47,49–51^. The AWEIsh and AWEInsh indices are effective at detecting and preserving water bodies with higher sediment loads, such as those with a high percentage of glacial rock flour^52^. In combination, the multi-spectral indices are an effective approach to successful water classification by utilising the strength of each index. After applying a threshold-based filtering to each of the indices, a series of post-processing stages were applied. These post-processing stages included raster-to-polygon conversion of the classified water bodies, applying an ocean mask, merging the overlapping polygon features, and applying a minimum area threshold of 0.05$\,{{\text{ km }}^{2}}$ to the merged polygons.

**Table 1:** Description of each of the spectral indices used in this study and their detection strengths.

Index	Expression (Sentinel-2 bands)	Strengths and target	References
NDWI (normalised difference water index)	$({\text{ B3 }} - {\text {B8}})/({\text {B3}} + {\text {B8}})$	Water and shadow, sediment-loaded water and snow/ice	McFeeters^53^
MNDWI (modified normalised difference water index)	$({\text {B3}} - {\text {B11}})/({\text {B3}} + {\text {B11}})$	Water and snow and ice	Xu^54^
AWEIsh (automatic water extraction index shadow)	${\text {B2}} + 2.5 * {\text {B3}} - 1.5 * ({\text {B8}} + {\text {B11}}) -0.25 * {\text {B12}}$	Sediment-loaded water	Feyisa et al.^52^
AWEInsh (automatic water extraction index no shadow)	$4 * ({\text {B3}} - {\text {B11}}) - (0.25 * {\text {B8}} + 2.75 * {\text {B12}})$	Sediment-loaded water	Feyisa et al.^52^
Brightness	$({\text {B4}} + {\text {B3}} + {\text {B2}})/3$	Snow covered areas	–

### ArcticDEM sink detection

Basins were classified from the ArcticDEM 10 m mosaic (Release 7, Version 3.0, https://www.pgc.umn.edu/data/arcticdem/) using a sink detection approach, commonly used for extracting large-scale topographic structures such as watersheds, streams and depressions^55^. The ArcticDEM is derived from high-resolution commercial optical stereo satellite imagery, generated through an adapted version of the automated Ames Stereo Pipeline^56,57^. The ArcticDEM mosaic is comprised of strip data acquired between 2009 and 2017, which is averaged, filtered and validated against filtered IceSAT altimetry data. Whilst the ArcticDEM mosaic does not represent a discrete time step, it was included in this study over the raw strip data because of the known limitations with using the strip data, namely limited accuracy, heavy reliance on validation datasets, and inhibited study feasibility^58,59^. Therefore, basins detected from the ArcticDEM mosaic reflect a temporal average and are not indicative of conditions at a specific time.

Sinks were defined by filling topographic depressions in the DEM to its pour point (i.e. the minimum elevation along its watershed boundary), following which the original DEM elevation value was subtracted from the depression-filled DEM elevation value^60^. Shallow sinks ($<5$ m deep, or $< 0.05$ $\,{{\text{ km }}^{2}}$ areal extent) were filtered to remove mis-classifications and water bodies with an insignificant water drainage volume, and limit the risk of discounting sinks that are consistently at, or exceeding, their pour point^25^.

### Dataset compiling

The water bodies derived from each approach (S1, S2 and ADEM) were combined and filtered in a semi-automated fashion. Water bodies were masked using a 1 km buffer around a modified version of the MEaSUREs GIMP 15 m ice mask (produced from a 1999 to 2001 image mosaic, https://nsidc.org/data/NSIDC-0714/versions/1)^33,61^. The ice mask was modified manually using coinciding Sentinel-2 imagery and a Landsat 8 image mosaic^62^ to update marked changes in the ice margin and outlet positions. Finally, the inventory was filtered and verified manually for quality purposes, as is standard practise in similar studies such as those looking at the Himalaya^63–65^. Detected features were removed from the inventory if they were not water filled or did not have any visible sign of drainage (such as waterline marks), based on manual validation against coinciding Sentinel-2 optical imagery. Following this, the dataset was populated with the appropriate metadata including detection method/s, basin location (based on those defined by Mouginot and Rignot^32^, https://nsidc.org/data/NSIDC-0714/versions/1), and lake name (provided by the Language Secretariat of Greenland, Oqaasileriffik, placename database).

### Error estimation

Error analysis was conducted to estimate the certainty of lake presence in the IIML (i.e. lake frequency). Four discrete 10,000$\,{{\text{ km }}^{2}}$ regions were selected to conduct the error analysis, covering the NE, NW and SW margins, and including the IC sector. Two independent users manually defined ice marginal lakes in each of these regions, using cloud-free Sentinel-2 imagery captured within the acquisition period that the IIML was derived (01/08-13/09/2017). The users defined an ice marginal lake using a single annotated point overlapping with the location of the lake on the Sentinel-2 image. The user-defined ice marginal lakes were compared to those from the IIML to determine differences in the number of lakes present in each region. A successful match is deemed as a user-defined point that is either overlapping or within 10 m of a polygon from the IIML. Each region in the error analysis had an average of 68 lakes present, with a maximum number of 100 lakes, as defined by the two users. There was minimal discrepancy between the users, with an average difference of three lakes per 10,000$\,{{\text{ km }}^{2}}$ region. User-defined lakes below a surface area of 0.05$\,{{\text{ km }}^{2}}$ were then removed to match the size threshold of the IIML. Overlap analysis was performed to determine corresponding lakes between the user-defined datasets and the IIML. Overall, the IIML captured 92% of the user-defined ice marginal lakes. This forms an error estimate for lake frequency in the IIML of $\pm 8$%, or $\pm 201$ lakes, as reported in the presented results.

## Supplementary Information

Supplementary Information.

## Data Availability

The 2017 inventory of ice marginal lakes is a data product under the ESA Glaciers CCI (Climate Change Initiative), freely available through the CEDA (Centre for Environmental Data Analysis) Archive at https://catalogue.ceda.ac.uk/uuid/7ea7540135f441369716ef867d217519.

## References

[1] IPCC. *IPCC Special Report on the Ocean and Cryosphere in a Changing Climate* (2019). 10.1007/s13280-019-01313-8 PMC7413947 31994026

[2] Mouginot J (2019). Forty-six years of Greenland Ice Sheet mass balance from 1972 to 2018. PNAS.

[3] Hanna, E. Greenland surface air temperature changes from 1981 to 2019 and implications for ice-sheet melt and mass-balance change. *Int. J. Climatol.* **41**, E1336–E1352. 10.1002/joc.6771 (2021).

[4] Shepherd A (2020). Mass balance of the Greenland Ice Sheet from 1992 to 2018. Nature.

[5] Lewis SM, Smith LC (2009). Hydrologic drainage of the Greenland Ice Sheet. Hydrol. Process..

[6] Haeberli W, Linsbauer A (2013). Brief communication “Global glacier volumes and sea level—Small but systematic effects of ice below the surface of the ocean and of new local lakes on land”. Cryosphere.

[7] Loriaux T, Casassa G (2013). Evolution of glacial lakes from the Northern Patagonia Icefield and terrestrial water storage in a sea-level rise context. Glob. Planet Change.

[8] Huss M, Hock R (2015). A new model for global glacier change and sea-level rise. Front. Earth Sci..

[9] Sutherland, J. L. *et al.* Proglacial lakes control glacier geometry and behavior during recession. *Geophys. Res. Lett.* **47**, e2020GL088865. 10.1029/2020GL088865 (2020).

[10] Shugar DH (2020). Rapid worldwide growth of glacial lakes since 1990. Nat. Clim. Change..

[11] Russell AJ, Carrivick JL, Ingeman-Nielsen T, Yde JC, Williams M (2011). A new cycle of jökulhlaups at Russell Glacier, Kangerlussuaq, West Greenland. J. Glaciol..

[12] Wolfe, D.F.G., Kargel, J.S. & Leonard, G.J. Glacier-dammed ice-marginal lakes of Alaska. in Kargel, J.S., Leonard, G.J., Bishop, M.P., Kääb, A. & Raup, B.H. (eds.) *Global Land Ice Measurements from Space, Springer Praxis Books* 263–295 (Springer, Berlin, 2014).

[13] Carrivick JL (2017). Ice-dammed lake drainage evolution at Russell Glacier, West Greenland. Front. Earth Sci..

[14] Carrivick JL, Tweed FS (2019). A review of glacier outburst floods in Iceland and Greenland with a megafloods perspective. Earth. Sci. Rev..

[15] Anderson NJ (2017). The arctic in the twenty-first century: Changing biogeochemical linkages across a paraglacial landscape of greenland. BioScience.

[16] Storms JEA, de Winter IL, Overeem I, Drijkoningen GG, Lykke-Andersen H (2012). The Holocene sedimentary history of the Kangerlussuaq Fjord-valley fill, West Greenland. Quat. Sci. Rev..

[17] Kjeldsen KK (2014). Ice-dammed lake drainage cools and raises surface salinities in a tidewater outlet glacier fjord, west Greenland. J. Geophys. Res. Earth Surf..

[18] Higgins AK (1970). On some ice-dammed lakes in Frederikshåb district, south-West Greenland. Medd. Dansk Geol. Foren..

[19] Weidick A, Citterio M (2011). The ice-dammed lake Isvand, West Greenland, has lost its water. J. Glaciol..

[20] Carrivick JL, Turner AGD, Russell AJ, Ingeman-Nielsen T, Yde JC (2013). Outburst flood evolution at Russell Glacier, western Greenland: Effects of a bedrock channel cascade with intermediary lakes. Quat. Sci. Rev..

[21] McGrath D (2010). Sediment plumes as a proxy for local ice-sheet runoff in Kangerlussuaq Fjord, West Greenland. J. Glaciol..

[22] Carrivick JL, Quincey DJ (2014). Progressive increase in number and volume of ice-marginal lakes on the western margin of the Greenland Ice Sheet. Glob. Planet. Change.

[23] Mernild SH, Hasholt B (2009). Observed runoff, jökulhlaups and suspended sediment load from the Greenland ice sheet at Kangerlussuaq, West Greenland, 2007 and 2008. J. Glaciol..

[24] Harrison S (2018). Climate change and the global pattern of moraine-dammed glacial lake outburst floods. Cryosphere.

[25] Larsen MAD (2013). A satellite perspective on jökulhlaups in Greenland. Hydrol. Res..

[26] Mallalieu J, Carrivick JL, Quincey DJ, Smith MW (2020). Calving seasonality associated with melt-undercutting and lake ice cover. Geophys. Res. Lett..

[27] Williamson AG, Arnold NS, Banwell AF, Willis IC (2017). A Fully automated Supraglacial lake area and volume Tracking (“FAST”) algorithm: Development and application using MODIS imagery of West Greenland. Remote Sens. Environ..

[28] Miles KE, Willis IC, Benedek CL, Williamson AG, Tedesco M (2017). Toward monitoring surface and subsurface lakes on the greenland ice sheet using sentinel-1 SAR and landsat-8 OLI imagery. Front. Earth Sci..

[29] Pope A (2016). Estimating supraglacial lake depth in West Greenland using Landsat 8 and comparison with other multispectral methods. Cryosphere.

[30] Leeson AA (2013). A comparison of supraglacial lake observations derived from MODIS imagery at the western margin of the Greenland ice sheet. J. Glaciol..

[31] Wangchuk S, Bolch T (2020). Mapping of glacial lakes using Sentinel-1 and Sentinel-2 data and a random forest classifier: Strengths and challenges. Sci. Remote Sens..

[32] Mouginot J, Rignot E (2019). Glacier catchments/basins for the Greenland Ice Sheet. UC Irvine Dataset..

[33] Howat, I. MEaSUREs Greenland Ice Mapping Project (GIMP) land ice and ocean classification mask, version 1 [GimpIceMask 15 m tiles 0-5]. *NASA National Snow and Ice Data Center Distributed Active Archive Center, Boulder, Colorado USA*. 10.5067/B8X58MQBFUPA (2017).

[34] Strozzi T, Wiesmann A, Kääb A, Joshi S, Mool P (2012). Glacial lake mapping with very high resolution satellite SAR data. Nat. Hazards Earth Syst. Sci..

[35] Hanna E (2008). Increased runoff from melt from the greenland ice sheet: A response to global warming. J. Clim..

[36] Mernild SH, Malmros JK, Yde JC, Knudsen NT (2012). Multi-decadal marine- and land-terminating glacier recession in the Ammassalik region, southeast Greenland. Cryosphere.

[37] Carrivick JL, Tweed FS, Sutherland JL, Mallalieu J (2020). Toward numerical modeling of interactions between ice-marginal proglacial lakes and glaciers. Front. Earth Sci..

[38] Catania GA, Stearns LA, Moon TA, Enderlin EM, Jackson RH (2020). Future evolution of Greenland’s marine-terminating outlet glaciers. J. Geophys. Res. Earth Surface.

[39] Christensen, T. R., Arndal, M. F. & Topp-Jørgensen, E. *Greenland Ecosystem Monitoring Annual Report Cards 2019* (Aarhus University, DCE—Danish Centre for Environment and Energy, 2020).

[40] Hopwood MJ (2020). Review article: How does glacier discharge affect marine biogeochemistry and primary production in the Arctic?. Cryosphere.

[41] Frey O, Santoro M, Werner CL, Wegmuller U (2013). DEM-based SAR pixel-area estimation for enhanced geocoding refinement and radiometric normalization. IEEE Geosci. Remote Sens. Lett..

[42] Wegmüller U (1999). Automated terrain corrected SAR geocoding. Proc. IGARSS’99.

[43] Wegmüller, U., Werner, C., Strozzi, T. & Wiesmann, A. Automated and precise image registration procedures. in *Analysis of Multi-Temporal Remote Sensing Images, Series in Remote Sensing*, vol. 2, 37–49 (World Scientific, 2002).

[44] Santoro M, Wegmüller U (2014). Multi-temporal synthetic aperture radar metrics applied to map open water bodies. IEEE J. Sel. Top. Appl. Earth Obs. Remote Sens..

[45] Santoro M (2015). Strengths and weaknesses of multi-year Envisat ASAR backscatter measurements to map permanent open water bodies at global scale. Remote Sens. Environ..

[46] Williamson AG, Banwell AF, Willis IC, Arnold NS (2018). Dual-satellite (Sentinel-2 and Landsat 8) remote sensing of supraglacial lakes in Greenland. Cryosphere.

[47] Chen F, Zhang M, Tian B, Li Z (2017). Extraction of glacial lake outlines in Tibet Plateau using landsat 8 imagery and google earth engine. IEEE J. Sel. Top. Appl. Earth Obs. Remote Sens..

[48] Burrough PA, McDonnell R, McDonnell RA, Lloyd CD (2015). Principles of Geographical Information Systems.

[49] Feng M, Sexton JO, Channan S, Townshend JR (2016). A global, high-resolution (30-m) inland water body dataset for 2000: First results of a topographic-spectral classification algorithm. Int. J. Digit. Earth.

[50] Huggel C, Kääb A, Haeberli W, Teysseire P, Paul F (2002). Remote sensing based assessment of hazards from glacier lake outbursts: A case study in the Swiss Alps. Can. Geotech. J..

[51] Bolch T, Buchroithner MF, Peters J, Baessler M, Bajracharya S (2008). Identification of glacier motion and potentially dangerous glacial lakes in the Mt. Everest region/Nepal using spaceborne imagery. Nat. Hazards Earth Syst. Sci..

[52] Feyisa GL, Meilby H, Fensholt R, Proud SR (2014). Automated Water Extraction Index: A new technique for surface water mapping using Landsat imagery. Remote Sens. Environ..

[53] McFeeters SK (1996). The use of the Normalized Difference Water Index (NDWI) in the delineation of open water features. Int. J. Remote Sens..

[54] Xu H (2006). Modification of normalised difference water index (NDWI) to enhance open water features in remotely sensed imagery. Int. J. Remote Sens..

[55] Jenson SK, Domingue JO (1988). Extracting topographic structure from digital elevation data for geographic information-system analysis. Photogramm. Eng. Rem. Sens..

[56] Shean DE (2016). An automated, open-source pipeline for mass production of digital elevation models (DEMs) from very-high-resolution commercial stereo satellite imagery. ISPRS J. Photogramm. Remote Sens..

[57] Porter C (2018). ArcticDEM. Hardvard Dataverse..

[58] Błaszczyk M (2019). Quality assessment and glaciological applications of digital elevation models derived from space-borne and aerial images over two tidewater glaciers of Southern Spitsbergen. Remote Sens..

[59] Bowling JS, Livingstone SJ, Sole AJ, Chu W (2019). Distribution and dynamics of Greenland subglacial lakes. Nat. Commun..

[60] Planchon O, Darboux F (2002). A fast, simple and versatile algorithm to fill the depressions of digital elevation models. CATENA.

[61] Howat IM, Negrete A, Smith BE (2014). The Greenland Ice Mapping Project (GIMP) land classification and surface elevation data sets. Cryosphere.

[62] Chen Z (2020). A new image mosaic of Greenland using Landsat-8 OLI images. Sci. Bull..

[63] Nie Y, Liu Q, Liu S (2013). Glacial lake expansion in the Central Himalayas by Landsat Images, 1990–2010. PLOS ONE.

[64] Aggarwal S, Rai SC, Thakur PK, Emmer A (2017). Inventory and recently increasing GLOF susceptibility of glacial lakes in Sikkim, Eastern Himalaya. Geomorphology.

[65] Shukla, A., Garg, P. K. & Srivastava, S. Evolution of glacial and high-altitude lakes in the Sikkim, Eastern Himalaya over the past four decades (1975–2017). *Front. Environ. Sci.* **6**, 81. 10.3389/fenvs.2018.00081 (2018).

[66] Esri Inc. ArcGIS Pro (v2.6.1) (2020).

