# Supplementary material for: Greenland-wide inventory of ice marginal lakes using a multi-method approach
Source: Sci Rep. 2021 Feb 24;11:4481. doi: 10.1038/s41598-021-83509-1 (PMC7904918; doi:10.1038/s41598-021-83509-1)
Supplement: Supplementary file 1 — Supplementary Information. [file 41598_2021_83509_MOESM1_ESM.pdf]

Supplementary Material: Greenland-wide Inventory of Ice Marginal Lakes using a multi-method approach

Penelope How[1*], Alexandra Messerli[1], Eva Mätzler[1], Maurizio Santoro[2], Andreas Wiesmann[2], Rafael Caduff[2], Kirsty Langley[1], Mikkel Høegh Bojesen[1†], Frank Paul[3], Andreas Kääb[4], and Jonathan L. Carrivick[5]

[1]Asiaq Greenland Survey, Nuuk, Greenland
[2]Gamma Remote Sensing, Gümligen, Switzerland
[3]Department of Geography, University of Zurich, Zurich, Switzerland
[4]Department of Geosciences, University of Oslo, Oslo, Norway
[5]School of Geography and water@leeds, University of Leeds, Leeds, UK
[†]Now at: DHI GRAS, Hørsholm, Denmark
[*]Corresponding author: how@asiaq.gl

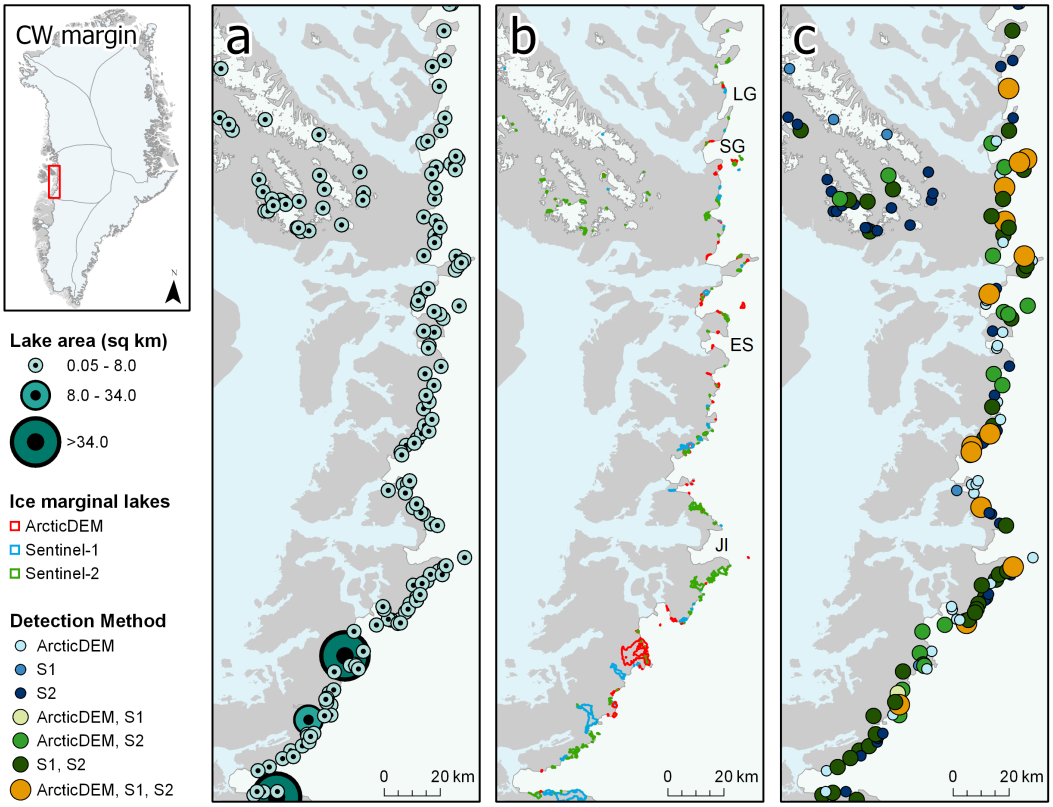

Figure S1. Ice marginal lakes over a selected section of the CW ice sheet margin, where (a) lake area, (b) lake shape determined by each method (as described in the Methods section on the manuscript), and (c) detection method are presented. Key outlets are illustrated in panel b, including Eqip Sermia (ES), Store Glacier (SG) and Lille Glacier (also known as Sermeq Avannarleq, LG). Ice, land and ocean are displayed in white, grey and light blue, respectively. The ice margin shown is a modified version of the MEaSUREs GIMP ice mask[1]. Figure generated with ArcGIS Pro (v2.6.1, https://www.esri.com/en-us/arcgis/products/arcgis-pro/)[2].

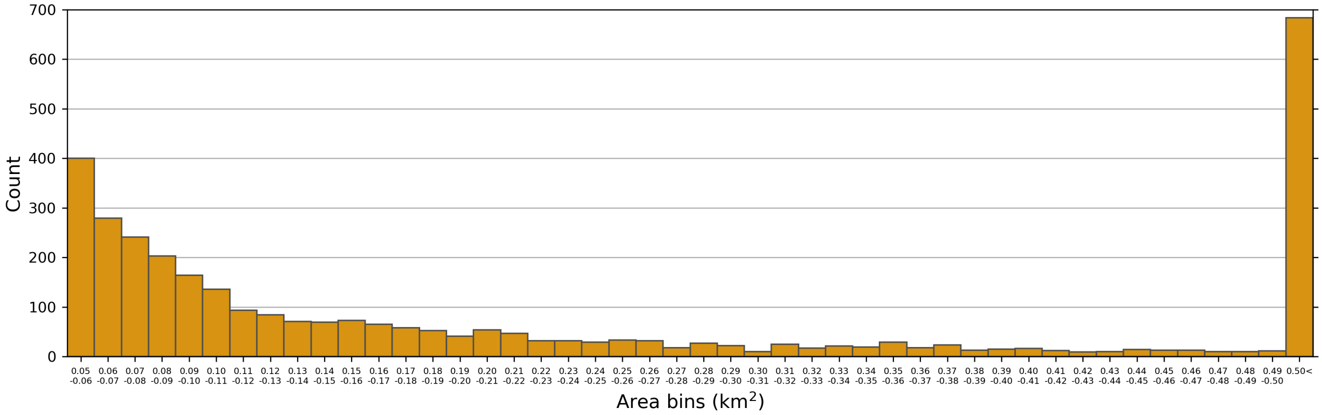

Figure S2. Histogram distribution of ice marginal lake area from the 2017 IIML (inventory of ice marginal lakes).

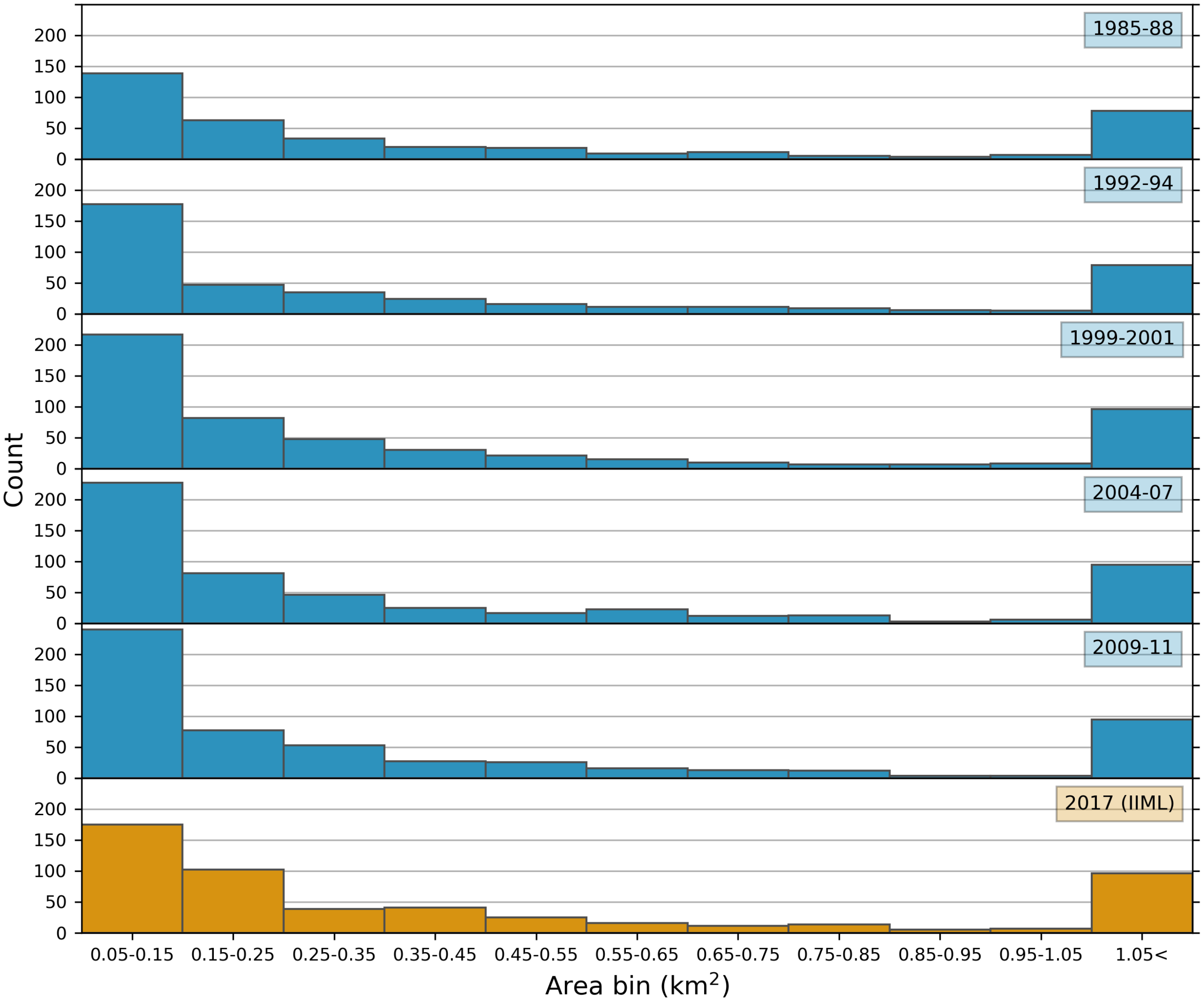

Figure S3. Histogram distribution of ice marginal lake area in West Greenland from 1985 to 2017, where the time steps from 1985 to 2014 (blue) are from Carrivick and Quincey (2014) and the 2017 time step is the coinciding subset from the 2017 IIML (inventory of ice marginal lakes).

Basin Name	Lake count	Lake area (km^2)					Detection methods						
		Sum	Average	Median	Mode	Std Dev	S1	S2	ADEM	S1,S2	S1,ADEM	S2,ADEM	All
CW	144	140.64	0.98	0.18	0.06	3.17	3	35	33	40	1	16	16
IC	948	492.25	0.52	0.13	0.06	2.04	156	273	337	105	11	51	15
NE	696	894.58	1.29	0.22	0.09	4.00	13	142	373	46	1	97	24
NO	247	363.87	1.47	0.23	0.07	5.76	4	86	100	7	0	47	3
NW	246	110.42	0.45	0.13	0.06	1.49	9	62	97	31	0	35	12
SE	385	151.78	0.39	0.09	0.06	1.63	51	58	218	22	3	29	4
SW	681	747.18	1.10	0.17	0.06	4.65	33	140	260	112	5	86	45

Table S1. IIML statistics by basin, including total lake area, individual lake area statistics and method performance.

Ice marginal lakes detected	Count
Total number of unique lakes	3347
Detected using one method	2483 (74%)
ArcticDEM	1418
S1	269
S2	796
Detected using two methods	745 (22%)
ArcticDEM and S1	21
ArcticDEM and S2	361
S1 and S2	363
From three sources	119 (4%)

Table S2. IIML detection method summary.

Methods comparison	Average extent difference	
	Absolute (km^2)	Percentage (%)
S1 vs. S2	0.14	47%
S1 vs. ADEM	0.36	64%
S2 vs. ADEM	0.25	49%
Maximum vs. minimum	0.37	70%

Table S3. Difference in lake extent between the three lake detection methods (S1, S2 and ADEM), performed on ice marginal lakes within the IIML that were detected successfully with all three methods.

References

1. Howat, I. MEaSUREs Greenland Ice Mapping Project (GIMP) Land Ice and Ocean Classification Mask, Version 1 [GimpIceMask 15m tiles 0-5]. *NASA Natl. Snow Ice Data Cent. Distributed Active Arch. Center, Boulder, Colo. USA* DOI: 10.5067/B8X58MQBFUPA (2017).
2. Esri Inc. ArcGIS Pro (v2.6.1) (2020).
